# Tyroviruses are a new group of temperate phages that infect *Bacillus* species in soil environments worldwide

**DOI:** 10.1186/s12864-022-09023-4

**Published:** 2022-11-28

**Authors:** Steven Batinovic, Cassandra R. Stanton, Daniel T. F. Rice, Brittany Rowe, Michael Beer, Steve Petrovski

**Affiliations:** 1 Department of Physiology, Anatomy, and Microbiology, La Trobe University, Bundoora, VIC Australia; 2 Present address: Division of Materials Science and Chemical Engineering, Yokohama National University, Yokohama, Kanagawa Japan; 3 Defence Science and Technology Group, Fishermans Bend, Victoria, Australia

**Keywords:** Bacteriophage, *Bacillus anthracis*, *Bacillus cereus*, Tyrovirus, Genomics

## Abstract

**Background:**

Bacteriophages are widely considered to be highly abundant and genetically diverse, with their role in the evolution and virulence of many pathogens becoming increasingly clear. Less attention has been paid on phages preying on *Bacillus*, despite the potential for some of its members, such as *Bacillus anthracis*, to cause serious human disease.

**Results:**

We have isolated five phages infecting the causative agent of anthrax, *Bacillus anthracis*. Using modern phylogenetic approaches we place these five new *Bacillus* phages, as well as 21 similar phage genomes retrieved from publicly available databases and metagenomic datasets into the Tyrovirus group, a newly proposed group named so due to the conservation of three distinct tyrosine recombinases. Genomic analysis of these large phages (~ 160–170 kb) reveals their DNA packaging mechanism and genomic features contributing to virion morphogenesis, host cell lysis and phage DNA replication processes. Analysis of the three tyrosine recombinases suggest Tyroviruses undergo a prophage lifecycle that may involve both host integration and plasmid stages. Further we show that Tyroviruses rely on divergent invasion mechanisms, with a subset requiring host S-layer for infection.

**Conclusions:**

Ultimately, we expand upon our understanding on the classification, phylogeny, and genomic organisation of a new and substantial phage group that prey on critically relevant *Bacillus* species. In an era characterised by a rapidly evolving landscape of phage genomics the deposition of future Tyroviruses will allow the further unravelling of the global spread and evolutionary history of these *Bacillus* phages.

**Supplementary Information:**

The online version contains supplementary material available at 10.1186/s12864-022-09023-4.

## Background

Bacteriophages (or phages) are bacterial viruses that are considered the most abundant biological entity in nature, with estimates suggesting the presence of 10^31^ phage particles on the planet at any one time [1, 2]. Phages selectively target and infect their bacterial host and hijack cellular machinery ultimately resulting in host lysis. They are thought to be implicated in maintaining an ecological balance in mixed communities and can influence the microbial balance by direct interactions with its host [2–4].

*Bacillus* are spore forming gram-positive bacteria commonly encountered in the soil environment. Two species of this genera, *Bacillus cereus* and *Bacillus anthracis* are known for their ability to cause human food borne illnesses and anthrax, respectively [5]. *B. anthracis* infection poses a high risk of serious illness or death to humans exposed, and is considered a national security risk given bioterrorism attacks have been previously reported using *B. anthracis* spores [6]. Due to the pathogenicity of *B. anthracis* and its potential use in bioterrorism, rapid detection of the living bacterium and its spores is essential. Dated detection methods of *B. anthracis* included culturing the organism and then differentiating it from other *Bacillus* species which resorts to the use of molecular techniques, which can take several days [7]. An attractive approach to detect live *B. anthracis* cells is using phages that specifically target their host. Indeed, the gamma phage assay, utilising the *Bacillus anthracis* phage γ, is a now widely used diagnostic test to detect *B. anthracis* in laboratories around the world owing to its ease and low cost of use [8]. *Bacillus* phage γ (and various γ-like phages such as Cherry and Fah) was first discovered in the 1950’s and was shown to be highly specific against both capsulated and non-capsulated variants of *B. anthracis*, the main driver behind its success as a diagnostic tool [9]. The *Bacillus* γ family of phages also forms the basis for some new generation potential diagnostics such as the engineering of phage for bioluminescent detection of *B. anthracis* [10, 11].

The preponderance of phage sequence data made available since the introduction of next generation sequencing has revealed an enormous phage genetic diversity. Understanding and resolving this diversity is a complex task given the large number of phages identified against model hosts. Some of the best studied phages that infect a single host are those infective for *Mycobacterium smegmatis* MC^2^ 155, for which almost 12,000 phages had been isolated, and over 2000 phages sequenced, at the time of writing [12, 13]. Based on their genome sequences the phages were categorised into different groups and clusters [14]. Similarly, bioinformatic analyses of 93 *Bacillus* phages resulted in the grouping of phages into 12 distinct clusters with 14 singleton phages [15]. One of these singleton phages was *Bacillus anthracis* phage Tsamsa, a large (169 kb) temperate phage with siphovirus morphology isolated in Namibia over 10 years ago [16]. Since then, multiple phages have been reported that share similar genome characteristics to Tsamsa phage [17–19].

In this study, five novel phages infecting *B. anthracis* were isolated from Australian soil samples. The phage genomes were sequenced and shown to be related to Tsamsa phage. Screening of the publicly available phage isolate sequences, and metagenome sequences revealed an additional 20 phages distributed globally that share similar genomic characteristics to Thrax1-5 and Tsamsa phages. Using comprehensive bioinformatics approaches we determine these phages belong to a new group we term the Tyrovirus group. Additionally, we analyse the morphology and genomic features of Tyroviruses and explore the nature of their interaction with the *Bacillus* host. This study ultimately expands a phage family, which was once a singleton, to now include an additional 25 phages under a new group, Tyrovirus, and delivers additional genomic insights and understanding of their evolutionary diversity.

## Results and discussion

### Isolation of five *Bacillus anthracis* phages

**Fig. 1:**
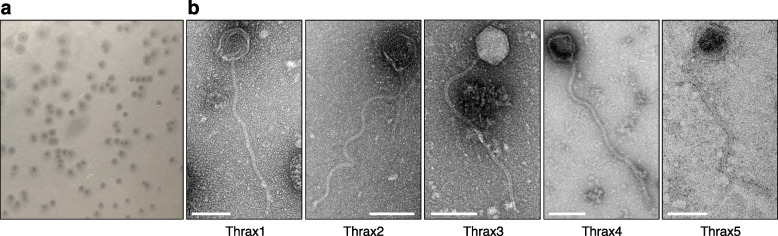
Isolation of five *Bacillus* phages, Thrax1-5. **a**, Plaques produced by *Bacillus* phage Thrax1 applied onto a *B. anthracis* lawn on solid medium. The image is representative of three biological replicates. **b**, Transmission electron microscopy of Thrax1-5 phages. Phage isometric icosahedral heads were measured to be approximately 78 ± 3 nm in diameter and the long non-contractile tails approximately 431 ± 28 nm in length Scale bars, 100 nm

Five temperate bacteriophages, Thrax1-5, were isolated from soil collected in Northern Territory, Australia in 2016. The phages were isolated on the avirulent *B. anthracis* strain Sterne (34F2) which lacks the pXO2 plasmid and the ability to generate the poly-γ-d-glutamatic acid capsule. Given the presence of this capsule has been shown to prevent infection by some *B. anthracis* phages [20], the lytic capacity of Thrax1-5 phages was also tested on nine capsulated (virulent) *B. anthracis* strains. Thrax1 phage caused host lysis on all nine strains while Thrax2, Thrax3 and Thrax5 phages produced lysis on only six, two and four strains, respectively (Supplementary Table 1). Phage virions negatively stained with uranyl acetate and imaged under transmission electron microscope displayed typical siphovirus morphology with isometric icosahedral heads approximately 78 ± 3 nm in diameter and long non-contractile tails approximately 431 ± 28 nm in length (Fig. 1).

### Phylogenetic positioning of Thrax phage genomes creates the Tyrovirus group

The phage isolates were sequenced using short-read Illumina technology leading to the assembly of five complete phage genomes. All five phages represent unique species (< 95% average nucleotide identity) and contain large 157–169 kb genomes with GC content ranging from 32–35% (Table 1) which is like that observed in their host, *B. anthracis* (35.4%), and other *B. cereus* group species.

**Table 1:** Characteristics of Tyroviruses

	**Size (bp)**	**GC %**	**CDS**	**tRNA**	**Host**	**Country**	**Source**	**Accession**	**Reference**
Thrax1	167,267	34.5	255	21	*B. anthracis*	Australia	Soil	ON548417	This study
Thrax2	164,774	33.4	248	17	*B. anthracis*	Australia	Soil	ON548418	This study
Thrax3	168,817	34.4	269	20	*B. anthracis*	Australia	Soil	ON548419	This study
Thrax4	169,483	34.4	267	22	*B. anthracis*	Australia	Soil	ON548420	This study
Thrax5	157,022	32.1	233	6	*B. anthracis*	Australia	Soil	ON548421	This study
Tsamsa	168,876	34.3	272	19	*B. anthracis*	Namibia	Animal	KC481682	[16]
PBC2	168,973	34.4	251	17	*B. cereus*	South Korea	Wastewater	KT070867	[17]
pW2	160,911	32.5	256^a^	5^a^	*B. cereus*	China	Wastewater	MK288021	[18]
Izhevsk	168,638	34.3	255	18	*B. cereus*	Russia	Soil	MT254578	[19]
Kirov	165,667	35.5	275	5	*B. cereus*	Russia	Soil	MW084976	
136	160,590	32.2	238	17	*B. cohnii*	Kenya	Soil	MH884508	[21]
MrDarsey	164,998	34.2	247	19	*B. anthracis*	USA	Wastewater	OK499987	
Sophrita	167,995	32.4	262	4	*B. anthracis*	USA	Wastewater	OK499991	
Nate	166,879	33.6	267	19	*B. anthracis*	USA	Soil	OK499992	
Chewbecca	161,151	34.3	237	20	*B. anthracis*	USA	Soil	OK499972	
Skywalker	160,928	34.3	236	21	*B. anthracis*	USA	Soil	OK499994	
PJN02	165,868	33.6	232^a^	13^a^	*B. subtilis*	China	Wastewater	OM634653	
FADO	164,756	33.6	243^a^	15^a^	*B. subtilis*	Portugal	Soil	OM236516	
Diildio	171,148	34.3	267^a^	21^a^	*B. thuringiensis*	USA	Soil	N/A^b^	
Tyro1	159,412	32.5	261^a^	6^a^	N/A	USA	Soil	Ga0207655_1000119^c^	3300025728^d^
Tyro2	139,829	35.3	219^a^	19^a^	N/A	Mexico	Plant	Ga0058696_1000002^c^	3300003846^d^
Tyro3	172,388	35.4	277^a^	19^a^	N/A	Mexico	Plant	Ga0209463_1000009^c^	3300027628^d^
Tyro4	155,345	33.7	231^a^	5^a^	N/A	USA	Soil	Ga0307505_1000004^c^	3300031455^d^
Tyro5	153,687	31.7	199^a^	3^a^	N/A	USA	Soil	Ga0207865_100044^c^	3300025529^d^
Tyro6	158,047	33.2	234^a^	8^a^	N/A	USA	Soil	Ga0193600_1000024^c^	3300018877^d^
Tyro7	160,694	33.2	241^a^	8^a^	N/A	USA	Soil	Ga0193606_1000028^c^	3300018839^d^

*NA* Not applicable

^a^ As determined by PROKKA

^b^ No accession number associated with this sequence in Bacillus Phage DB

^c^ IMG/VR scaffold ID

^d^ IMG/VR Genome ID

We examined public sequence databases for sequenced genomes similar to Thrax1-5 phages. Related cultivated phages included Tsamsa [16], Nate, Chewbecca, Skywalker, MrDarsey and Sophrita phages isolated on *B. anthracis*, PBC2 [17], pW2 [18], Izhevsk [19], and Kirov phages isolated on *B. cereus,* Diildio phage isolated on *B. thuringiensis*, FADO and PJN02 phages isolated on *B. subtilis*, and phage 136 isolated on *B. cohnii* [21]. These phages were mostly isolated from soil samples (except for Tsamsa phage isolated from an animal carcass, and MrDarsey, Sophrita, PBC2, pW2 and PJN02 phages from wastewater) from the USA, China, South Korea, Namibia, Kenya, Portugal and Russia between 2010–2021 (Table 1). Subsequent investigation of uncultivated viral genome assemblies from metagenome datasets using IMG/VR [22] identified seven additional unique phage genomes (estimated to be near-complete) with similarity to the above cultivated phage genomes, which we refer to as Tyro1-7 phages (Table 1). These phage genomes were predominantly found from soil and plant-associated microbiome analyses in Mexico and the USA.

**Fig. 2:**
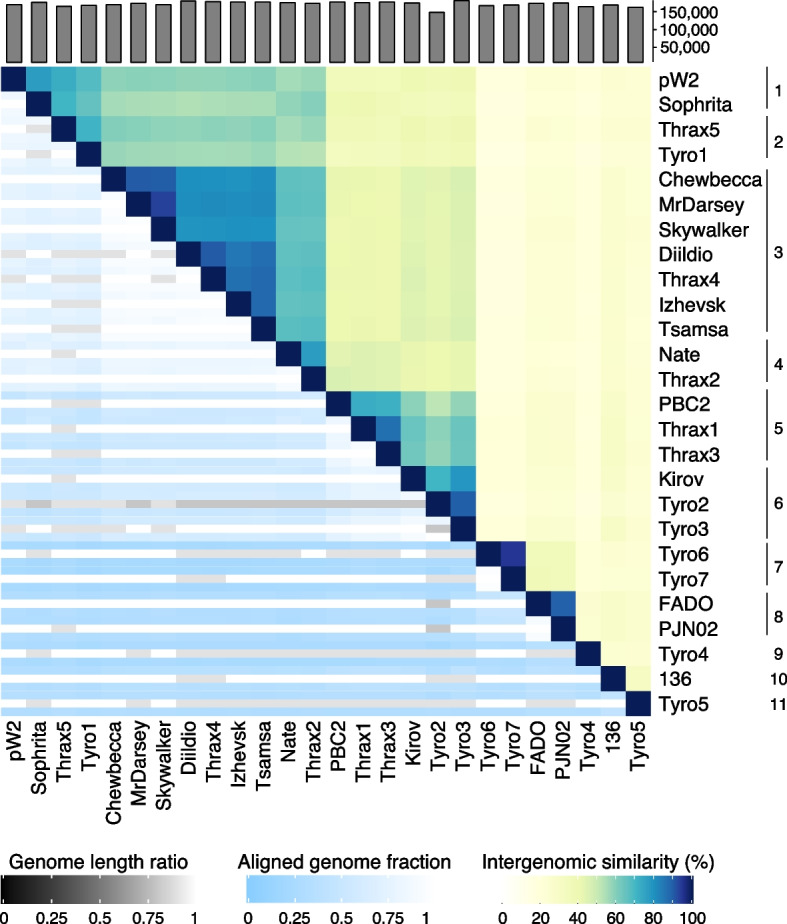
Whole genome nucleotide similarity between “Thrax-like” phages. A nucleotide similarity heatmap between the 26 “Thrax-like” phages demonstrating splitting into 11 genera groupings (based on ≥ 70% intergenomic similarity to cluster). Intergenomic similarities are shown on top/right hand side (scale from 0–100%) and aligned genome fraction (0–1) and genome length ratio (0–1) are shown on bottom/left side. Total genome size is shown in bars on top. Raw data are shown in Supplementary Table 2

Analysis of these 26 “Thrax-like” phages with VIRIDIC [23] revealed grouping into 11 genus clusters based on nucleotide similarity (≥ 70% intergenomic similarity; Fig. 2, Supplementary Table 2). Intergenomic similarity ranged from as high as 94.9% (Tyro6 vs Tyro7), just below the intra-species boundary (≥ 95%), to as low as 15.7% (Tyro1 vs Tyro7), despite all “Thrax-like” phages sharing equivalent genome sizes and remarkably similar genomic architectures (discussed later). Clustering was found to be independent to the geography of isolation and instead clustering showed tendency to correlate to host species (i.e. *B. subtilis* phages PJN02 and FADO, isolated from China and Portugal, respectively, formed a genus cluster).

**Fig. 3:**
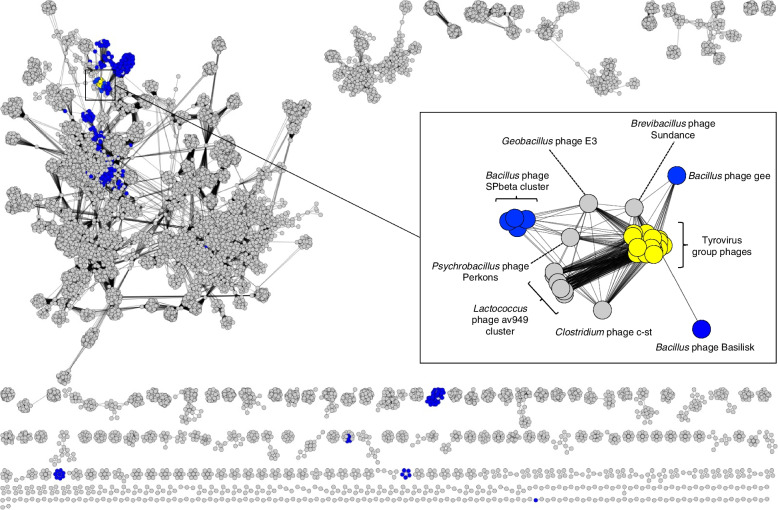
Tyroviruses cluster tightly using a reticulate phage genome network. **a**, A reticulate genome network of > 17,000 phage genomes sourced from the NCBI Genbank database. Individual phages, are represented by circular ‘nodes’, and phage relationships are illustrated by line ‘edges’. *Bacillus* phages are colored in blue and Tyroviruses are colored in yellow. Zoom panel shows tight clustering of Tyroviruses using an edge-weight spring embedded layout model. Raw viral cluster information is shown in Supplementary Table 3

To phylogenetically place and classify this group of phages, we made use of two divergent viral taxonomy approaches, namely vContact2 and VICTOR. vContact2 is a network-based strategy that computes viral clusters (VC) based on the degree of gene-sharing between phage genomes [24]. For this analysis, we input all complete phage genome sequences present in the GenBank database as of April 2022 (17,009 phage sequences) in addition to the 26 Thrax-like phage genomes above. Within the resulting network, the Thrax-like phages (Fig. 3; yellow nodes), cluster tightly together, near other cultivated phages that infect *Bacillus* (Fig. 3; blue nodes) and related genera. Nodes representing Thrax-like phages and neighbouring phages were extracted and visualised using an edge-weighted spring layout to better observe grouping characteristics (Fig. 3). Thrax-like phages clustered tightly together with closest phage neighbours identified as *Geobacillus* phage E3 and *Brevibacillus* phage Sundance, among others (Fig. 3; Supplementary Table 3). We have termed these 26 tightly clustered Thrax-like phages the Tyrovirus group, named due to the conservation of three tyrosine recombinases (discussed later). Tyroviruses grouped into an exclusive VC which was further defined into 16 sub-VC’s, with the largest sub-VC comprising Tsamsa, Izhevsk, Diildio and Thrax4 phage. (Supplementary Table 3). As Sub-VC’s are suggested to be approximately equivalent to genera [24], these groupings were broadly consistent with those calculated by VIRIDIC (Supplementary Figure. 1).

**Fig. 4:**
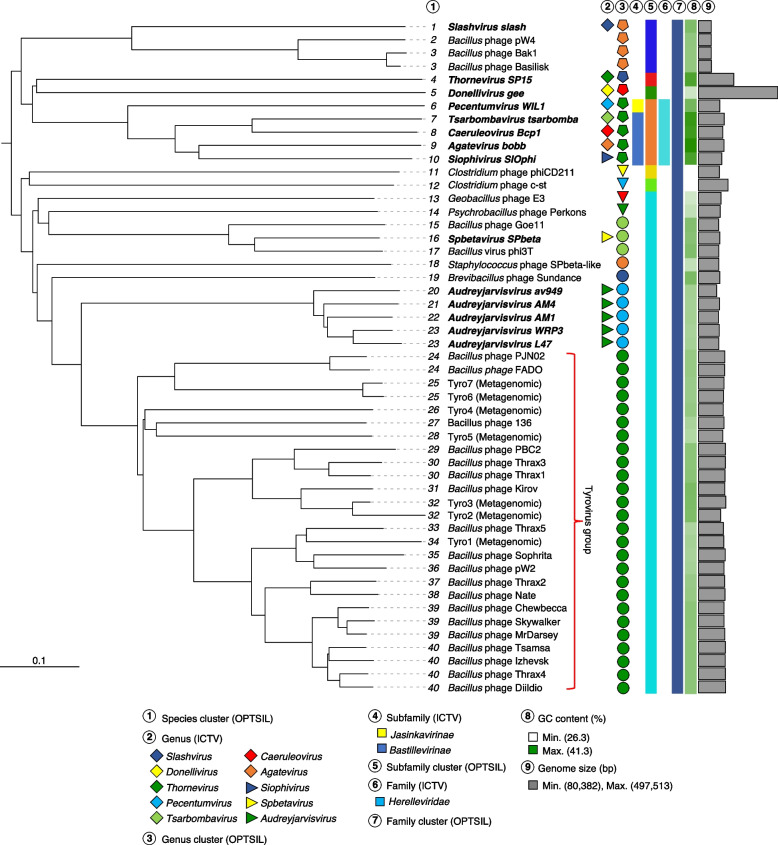
Phylogenetic placement of Tyroviruses. Whole genome-based phylogeny (amino acid) was inferred using Genome-BLAST Distance Phylogeny (100 bootstrap) using formula D6 yielding average support of 70%. OPTSIL clustering yielded 40 species clusters, 13 genus clusters, 7 subfamily clusters and 1 family cluster. Phages classified by the ICTV are noted in bold. Proposed Tyrovirus grouping is shown. Scale bar represents the number of amino acid substitutions per site

VICTOR relies on whole-genome phylogeny to determine evolutionary relationships between phages [25]. Here, input consisted of the total protein sequences of Tyroviruses and 25 related phages (deduplicated at < 95% nucleotide identity). VICTOR phylogeny combined with OPTSIL taxon boundary prediction suggested that Tyroviruses form a genus level clade (Fig. 4). This prediction is more aggressive than those suggested by VIRIDIC and vContact2 (which grouped the Tyroviruses into multiple genera/sub-VC’s). The nucleotide similarities between many of these phages fall below the ~ 70% threshold recommend by International Committee on Taxonomy of Viruses ICTV for genus-level grouping. While VICTOR utilises an optimised Genome Blast Distance Phylogeny (GBDP) to infer phylogenetic trees and has been optimised against a large reference phage genome database recognised by the ICTV [25], we are hesitant to suggest any genus-level classifications for laboratory isolated Tyroviruses given the inconsistencies between these analyses. Here we instead simply continue to refer to them, and the additional metagenome-derived phages, as Tyroviruses. When expanding out to subfamily predictions, OPTSIL grouped Tyroviruses with *Lactococcus* av949-like phages, *Brevibacillus* Sundance phage, *Bacillus* SPbeta-like phages and *Geobacillus* E3 phage (Fig. 4), which were similarly co-located in clusters via vContact2-derived network phylogeny (Fig. 3).

We next performed a pangenome assessment to determine the core proteome of Tyroviruses. Using a less stringent identity threshold of 30% [26], a set of 67 proteins (~ 25% total proteome) were identified to be in common across the 19 complete Tyrovirus genomes (i.e. excluding metagenomic assemblies from IMG/VR; Supplementary Fig. 1, Supplementary Table 4). This somewhat underestimates the similarities shared between these phages given this core proteome is encoded for by almost half (~ 45%) of the Tyroviral nucleotide sequence. The genetic elements of the core genome are represented by both early-stage effectors; 19 proteins in DNA replication/repair and nucleotide metabolism, and late-stage components; 15 proteins involved in virion morphogenesis and host lysis. The remainder of the core proteome included 5 proteins in DNA recombination (including three tyrosine recombinases) and 28 proteins of unknown function or hypothetical proteins.

### Genomic organisation

#### Genome termini

The precise genome ends of only a single Tyrovirus have been empirically determined to date. Tsamsa phage was shown to contain short direct terminal repeats (DTRs) of 284 bp [16]. To determine the genome termini of Thrax1-5 phages we used PhageTerm [27], in combination with manual inspection of the raw sequencing reads. Similar to that seen in Tsamsa phage, phages Thrax1-3 were found to contain 285 bp DTRs and phages Thrax4-5 contained 284 bp DTRs (Supplementary Fig. 2). The presence of short DTRs indicate these phages undergo T7 phage-like DNA packaging process where the in vivo DNA substrate for packaging is a concatemer of the phage DNA [28]. We proceeded to identify the genome ends of other isolated Tyrovirus phages based on sequence identity to the DTRs observed in Tsamsa phage and phages Thrax1-5. PBC2, pW2, Izhevsk, Kirov, Diildio, Nate, Chewbecca, Skywalker, MrDarsey and Sophrita phages were predicted to contain sequences consistent with the above empirically confirmed DTRs and show some areas of high sequence identity (Supplementary Fig. 2). We were unable to confirm the presence of DTRs in 136, PJN02 and FADO phages due to lower nucleotide similarity shared with the above phages. Using sequence identity of the phage terminase as a proxy for determining genome packaging strategy [28], we compared the terminase present in these three phages to the GenBank non-redundant protein database. Highest sequence similarity was observed to the terminase present in each other’s genomes, other Tyrovirus genomes, and to the terminase of *Brevibacillus* phage Sundance (~ 46% similarity). The packaging strategy of Sundance phage involves short DTRs (323 bp) [29], which in combination, highly suggests 136, PJN02 and FADO phages also employ short DTRs.

#### Modular structure

**Fig. 5:**
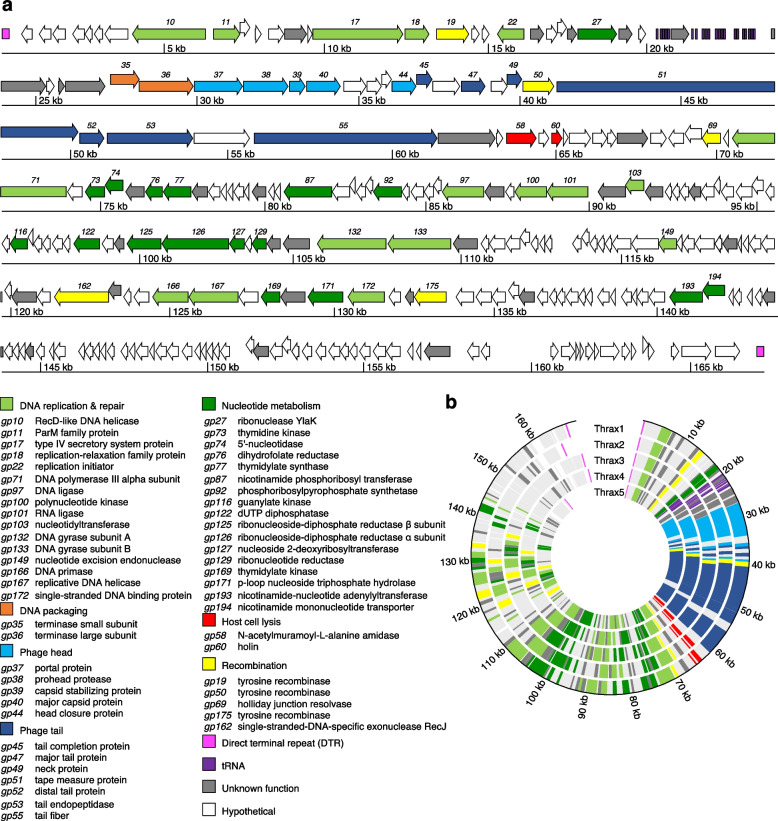
*Bacillus* phages Thrax1-5 contain large modular genomes. **a**, Genome map of Thrax1 illustrating its modular arrangement and large complement of DNA replication & repair and nucleotide metabolism genes. Annotated genes with known function are listed below the map. Genes required for phage DNA packaging, virion morphogenesis and host cell lysis are shown. Genes involved in recombination (including the three tyrosine recombinases) are shown. Furthermore, direct terminal repeats on either side of the genome and tRNAs are also shown. Unknown function indicates genes that contain a conserved domain but have no assignable function in the phage lifecycle. Hypothetical indicate genes that do not contain any conserved domains. **b**, Circular map comparing Thrax1-5 phages demonstrates similar genome organization. Size markers are shown in kb. Genome annotations are shown in Supplementary Tables 5–9

The Thrax1-5 phages have large linear genomes between 157–169 kb which encode between 233–268 ORFs, ~ 70% of which encode for hypothetical proteins, and between 6–22 tRNAs (Supplementary Tables 5–9). The genomes are characteristically organised into functional modules (Fig. 5) with the two more prominent modules (a “left arm” and “right arm”) encoding genes involved in virion morphogenesis and lysis (*gp35-66* in Thrax1) and replication and nucleotide metabolism, respectively (*gp67-241* in Thrax1).

#### Virion morphogenesis and lysis

**Fig. 6:**
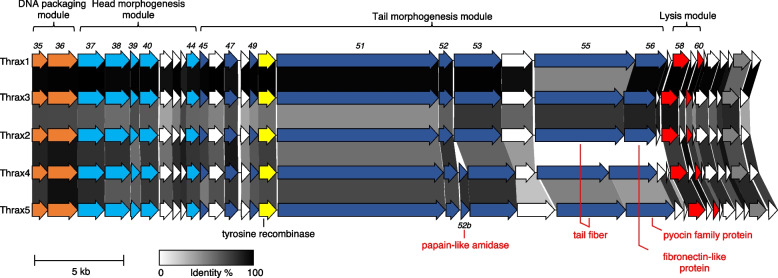
The virion morphogenesis and lysis module in *Bacillus* phages Thrax1-5. Module alignment between Thrax1-5 phages demonstrates high gene synteny and identity (shown by greyscale shading) in genes encoding the terminase subunits, phage capsid genes and host cell lysis genes. The phage tail genes are less conserved with Thrax4-5 containing an additional gene, papain-like amidase (*gp52b*), a different tail fiber *(gp55*)*,* and a pyocin family protein instead of a fibronectin-like protein (*gp56*) when compared to Thrax1-3. Gene color scheme is consistent with that described in Fig. 5. Text in red indicates gene differences between Thrax1-5. Shown for reference is one of the tyrosine recombinases (*gp50*). Scale bar represents 5 kb

The left arm contains 32 (Thrax1-3) or 34 (Thrax4-5) genes transcribed in the forward direction, most of which involved in the production, packaging, and release of phage progeny particles via host cell lysis (Fig. 5). The DNA packaging module, consisting of the first two genes in the arm, is well conserved among Thrax1-5 (91.9% average a.a. similarity; Fig. 6), encoding the terminase small and large subunits, respectively (Fig. 6; *gp35-36* in orange). The terminase enzyme is essential for packaging of the phage DNA into the phage head [30]. As an enzyme complex, the small subunit determines the specificity of DNA binding while the large subunit mediates the cleavage and packaging of phage DNA [30]. Both genes lacked identifiable conserved functional domains via the NCBI Conserved Domain Database (CDD) [31]. The small subunit was instead predicted based on 1), its genomic location (immediately upstream and encoded in the same direction as the large subunit) and 2), profile Hidden Markov model (HMM) similarity (via HHpred) [32] to known DNA restriction endonucleases and DNA-binding proteins, consistent with DNA-binding functionality shown in the terminase small subunit of *Escherichia* T4 phage [33]. The large subunit, outside other Tyrovirus group phages, showed highest similarity to the terminase large subunit of *Lactococcus* av949-like phages on GenBank and profile HMM similarity to that of the terminase large subunit in T4 phage [34].

The phage head morphogenesis module is located immediately downstream of the DNA packaging module (Fig. 6; *gp37-44* in light blue). These genes encode enzymatic and structural components required to assemble the phage capsid and were also well conserved across Thrax1-5 (82.6% average a.a. similarity). Many of these genes lacked identifiable conserved domains via CDD and were classified in combination with results from HHpred, and the Virfam webserver [35]. The first gene encodes the portal protein which forms a channel that works in tandem with the terminase complex to translocate DNA into the procapsid interior [30]. The next three genes encode the prohead protease, capsid stabilizing protein and major capsid protein (MCP), respectively (Fig. 6), the latter two assemble to form the icosahedral protein lattice called the procapsid [36]. The remaining four genes in this module consist of three hypothetical proteins with the remaining gene encoding the head closure protein which participates in the portal channel closure following DNA translocation [35].

The tail morphogenesis module follows (Fig. 6; *gp45-56* in dark blue) and encodes structural components of the large tail structure observed in the Thrax phages (Fig. 1). Genes were again classified based on results from CDD, HHpred and Virfam. The first three genes encode the tail completion protein, a hypothetical protein and the major tail protein (MTP), respectively. During phage assembly, copies of the MTP assemble to form the main tail tube which is then connected to the head via the tail completion protein [35, 37]. The next three genes include a hypothetical protein, the neck protein, and a tyrosine recombinase (discussed later). The neck protein is responsible for the association of the head and tail following successful packaging of viral DNA [35]. The next gene in this module encodes the tape measure protein which ranges from 3032–3150 a.a in Thrax1-5 phages. The length of the tape measure protein is known to correlate with the size of the assembled phage tail, here estimated to be between 455–473 nm (~ 0.15 nm per a.a [38]) which compares favourably to phage tail length measured using TEM (Fig. 1). The final cluster of 5–6 genes in this module showed the most divergence between the Thrax phages and could be broadly separated into two groups (Thrax1-3 and Thrax4-5). In the former, a distal tail protein, tail endopeptidase, tail fiber and fibronectin-like protein are encoded in this section (Fig. 6; *gp52*-*56*) while in the latter, this section additionally encodes a papain-like amidase (Fig. 6; *gp52b*) and a pyocin family protein instead of the fibronectin-like protein (Fig. 6; *gp56*). The sequence of tail fiber protein differs substantially between the two groups (29.0% cross-group average a.a. similarity). In the assembled phage virion, many of the tail proteins participate directly with the host during phage adsorption and invasion. The tail fiber is known to be a determinant for infection specificity via precise interaction with host receptors [39], while tail endopeptidases, permuted papain-like amidases and pyocins are involved in host cell wall degradation during initial stages of infection [40–43].

The final prominent cluster of identifiable genes in the left arm is the lysis module (Fig. 6; *gp58-60* in red). An N-acetylmuramoyl-L-alanine amidase (*gp58*) and holin (*gp60*) were classified based on results from CDD and were highly conserved between Thrax1-5 phages (90.9% average a.a similarity). Both lysis proteins work in concert to mediate phage virion release; holins form multimeric structures that puncture the bacterial membrane allowing N-acetylmuramoyl-L-alanine amidases to cleave the link between peptidoglycan found in host peptidoglycan causing cell lysis and phage virion release [44].

Further expanding our observation to all Tyroviruses demonstrated high conservation to genes within virion morphogenesis and lysis arm in agreement with the pangenome analysis showing ~ 70% of genes within this arm form part of the core proteome (Supplementary Fig. 1). As was the case in the analysis between Thrax phages, the largest variance across Tyroviruses were identified in the tail-encoding genes, with high divergence seen in the tail fiber gene (*gp55*). Given the role of the tail fiber in host specificity and adhesion one might expect these dissimilarities to correlate with differences in phage host range. Indeed, tail fibers from phages targeting *B. subtilis* (FADO and PJN02 phages) and *B. cohnii* (phage 139) showed phylogenetic branching away from the other phages (Supplementary Fig. 3). Tail fibers present in the remaining phages (which were isolated on *B. anthracis*, *B. cereus* and *B. thuringiensis*) did not clearly segregate according to host isolate, but seemed to broadly separate into two distinct groups, like that observed above with Thrax1-3 and Thrax4-5 (Fig. 6). This was not unsurprising given the closer relationship between these three *Bacillus* species and may indicate the ability of these phages to infect multiple species in the *Bacillus cereus* group, as was shown for Tsamsa phage [16]. Even so, these differences suggest the potential for variabilities in infection mechanism via interaction with different structures on the surface of the host (explored later). For host cell lysis, the N-acetylmuramoyl-L-alanine amidase was less conserved (73.6% average a.a similarity) than the holin (87.0% average a.a similarity) across Tyroviruses, especially in those endolysins found in phages isolated against *B. subtilis* and *B. cohnii*. Despite this, purified endolysin from Tsamsa, PBC2 and Izhevsk phages have been shown to lyse numerous *Bacillus* species as well as some species from *Prestia* (containing previous members previously of *Bacillus*), *Listeria* and *Clostridium* genera indicating broad range bacteriolytic activity [16, 17, 19].

#### DNA replication and nucleotide metabolism

The right arm contains between 154–195 genes (in Thrax1-5) transcribed in the reverse direction involved in DNA replication, repair, and nucleotide metabolism (Fig. 5; Supplementary Tables 5–9). Genes involved in DNA replication & repair were classified based on results from CDD and include DNA polymerase III alpha subunit (*gp71*), DNA ligase (*gp97*), polynucleotide kinase (*gp100*), RNA ligase (*gp101*), nucleotidyltransferase (*gp103*), DNA gyrase subunits A and B (*gp132, gp133)*, nucleotide excision repair endonuclease (*gp*149), DNA primase (*gp166*), replicative DNA helicase (*gp167*) and single-stranded DNA-binding protein (*gp172*; Fig. 5, light green). These genes are present in all five Thrax phages and are relatively well conserved (76.1% average a.a. similarity). The increased complement of DNA replication genes present in Thrax phages reduces the level of dependence of the phage on the host and suggests the phage optimises its DNA replication by utilising some phage-specific components. Interestingly, Thrax2, Thrax4 and Thrax5 were found to contain an additional RNA ligase in close proximity to the other. Phage-encoded RNA ligases, with an accompanying polynucleotide kinase, function in RNA remodelling and repair. In *E. coli* T4 phage, expression of these two proteins is well characterised to counter a host defence mechanism (RNA restriction by the host anticodon nuclease, PrrC) that depletes the available pool of functional tRNA^Lys^ in an attempt to prevent phage propagation [45, 46].

Nucleotide metabolism is critical to the function of DNA replication and repair. We additionally identified genes within the right arm that encode products involved in the metabolism of nucleotides (Fig. 5, dark green). These include thymidine, guanylate, and thymidylate kinases (*gp73, gp116, gp169*), 5’-nucleotidase (*gp*74), dihydrofolate reductase (*gp76*), thymidylate reductase (*gp77*), nicotinamide phosphoribosyl transferase (*gp87*), phosphoribosylpyrophosphate synthetase (*gp92*), dUTP diphosphatase (*gp122*), ribonucleoside-diphosphate reductase subunits alpha and beta (*gp125, gp126*), nucleoside 2-deoxyribosyltransferase (*gp127*), ribonucleotide reductase (*gp129*), p-loop-containing nucleoside triphosphate hydrolase (*gp171*), nicotinamide-nucleotide adenylyltransferase (*gp193*) and nicotinamide mononucleotide transporter (*gp194*). Not all these genes were found across all five Thrax phages (i.e. nicotinate salvage pathway components *gp87* or *gp193/gp194* were absent in some phages), however, we did note that most essential components involved in purine and pyrimidine biosynthesis were equivalently found in Thrax1-5.

Six genes encoding DNA replication & repair, and nucleotide metabolism, were also identified towards of the front of the Thrax phage genome (Fig. 5). The genes include a RecD-like DNA helicase (*gp10*), parM family protein (*gp11*), type IV secretion system protein (*gp17*), replication-relaxation family protein (*gp18*), replication initiator (*gp22*) and ribonuclease YlaK (*gp27*). Interestingly, the proteins encoded within the region from *gp11-18* resemble those seen in plasmids. ParM (*gp11*) is a nucleotide-driven motor protein involved in plasmid partitioning and commonly works in concert with a DNA-binding protein with a ribbon-helix-helix (RHH) motif often located immediately downstream of ParM [31]. Exploration of the gene downstream of *gp11* (*gp12 –* hypothetical protein) showed distant similarity to a ParG-like protein with an RHH motif. Type IV secretion proteins (*gp17*) are commonly involved in the mobilisation of conjugative plasmids while the replication relaxation protein (*gp18*) is essential for plasmid DNA replication. We believe this region (*gp11-18*) was transferred to an ancestral Tyrovirus phage (possibly from a conjugative plasmid) which is supported by the specific absence of *gp11-18* in the more distantly related Tyro6-7 phages and Tyrovirus-like prophages found in some *B. licheniformis*, *B. sonorensis* and *B. velezensis* strains (Supplementary Fig. 4).

Phages with larger genomes, such as Tyroviruses, tend to encode more DNA replication and nucleotide metabolism genes which should reduce their dependence on their host [47]. Indeed, we observe Tyroviruses encode many of their own tRNAs to further this independence which may also be used to avoid any host tRNA-cleavage self-defence mechanisms [48]. Some, but not all, Tyroviruses also encode capacity for biosynthesis of NAD^+^ via nicotinate salvage pathways which is required as a substrate for phage DNA replication [49]. However, despite the large number of DNA replication genes Tyroviruses would not display complete autonomy and still rely on host factors (i.e. host RNA polymerase) to complete their lifecycle.

#### Tyrosine recombinases

An unusual (and the namesake) feature of Tyroviruses is the presence of three distinct conserved tyrosine recombinases. Tyrosine recombinases are essential in the phage life cycle, with phage-encoded recombinases often involved in lysogeny via genome integration [50], or via the formation of circular intermediates by aiding in dimer resolution recognising and binding to repeats within an attachment site (*att*). The *att* is often represented by a short sequence flanked by repeat sequences. The repeat sequences are specifically recognised and bound by a tyrosine recombinase (either the same recombinase or two different recombinases to form homo- or heterodimers) which mediate processes such as scission post-chromosomal or plasmid DNA replication, or phage lysogeny [51]. In Tyroviruses, three genes (*gp19*, *gp50* and *gp175;* Fig. 5; Supplementary Tables 5–9) encode proteins with conserved motifs of the bacterial Xer tyrosine recombinases via CDD, with *gp19* containing a XerD domain, *gp175* a XerC domain, and *gp50* containing a dual purpose XerC and XerD domain. *Psychrobacillus* phage Perkons and *Vibrio vulnificus* phage pVv01 (published as a plasmid prophage) were some of the only phage sequences we could identify in public sequence databases also containing three tyrosine recombinases [52].

**Fig. 7:**
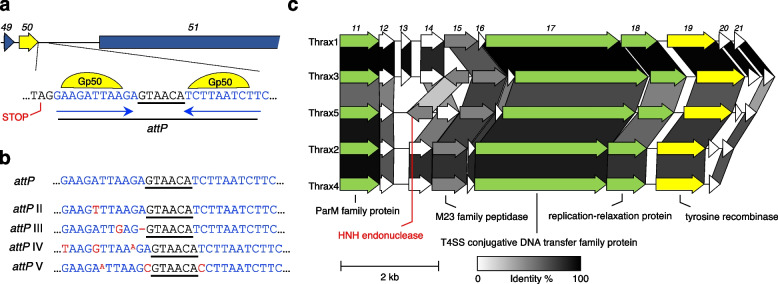
All Tyroviruses contain three distinct tyrosine recombinases. **a**, A putative phage attachment site (*attP*) discovered immediately downstream of the tyrosine recombinase (*gp50*). The *attP* features two 11-bp perfect inverted repeats, bound by Gp50 during recombination, which surround a 6-bp core sequence. **b**, Sequence conservation of the *attP* site in other Tyroviruses. Putative *attP* sequences (*attP* II-V) from other Tyroviruses were aligned and show minor discrepancies in the inverted repeat sections compared to the canonical *attP* detected in six phages (Thrax1, Thrax3, Kirov, PBC2, Tyro2 and Tyro3). All *attP* sequences are shown in Supplementary Table 10. **c**, Gene module alignment between Thrax1-5 phages including plasmid-like genes and another tyrosine recombinase (*gp19*). High gene identity (shown by greyscale shading) is noted across this region in Thrax1-5, and other Tyroviruses (except Tyro6-7; see Supplementary Fig. 4), suggesting a plasmid prophage lifecycle. Gene color scheme is consistent with that described in Fig. 5. Text in red indicates gene differences between Thrax1-5. Scale bar represents 2 kb

*Gp50* appears within the structural module located between the neck protein and tape measure protein (*gp49* and *gp51*, respectively; Fig. 7a). In addition to the XerC/XerD-like domain present in *gp50*, N-terminal phage integrase domain was also detected with CDD, suggesting it may function specifically as a phage integrase. To this end we analysed the sequence in the vicinity of *gp50* to see if there was an obvious phage attachment site (*attP*). A putative attachment site was found immediately downstream of the stop codon of *gp50* in Thrax1 featuring two 11-bp perfect inverted repeats surrounding a 6-bp core sequence site (Fig. 7b). The putative *attP* was remarkably well conserved among Tyroviruses known to infect *B. anthracis* and *B. cereus* species, but less so in those phages infecting *B. subtilis* or *B. cohnii* (Fig. 7b; Supplementary Table 10). It is difficult to determine the function of tyrosine recombinases based on sequence analysis and therefore hard to ascertain the function of *gp50* in relation to Tyroviruses lifecycle. The location of *gp50* in the structural module is especially curious, a feature shared with a tyrosine recombinase present in *Psychrobacillus* phage Perkons and the *Lactococcus* av949 family of phages, suggesting *gp50* is co-transcribed with other structural genes during the lytic cycle, contrary to a role in phage lysogeny. Further work is required to understand the specific role of *gp50* in Tyroviruses.

The remaining two tyrosine recombinases, *gp19* and *gp175*, are located near genes involved in DNA replication, albeit on opposite ends of the genome. Both contain either XerC or XerD domains, which are often responsible for dimer resolution of bacterial chromosomes and plasmids during cell division [53]. Interestingly, *gp19* is located next to the cluster of genes involved in plasmid maintenance (*gp11-gp18;* Fig. 7c). It is possible these genes support a plasmid prophage lifecycle, and thus the three recombinases within Tyroviruses could be responsible for facilitating a complex prophage lifecycle involving both host genome integration and maintenance of plasmid prophage.

#### Exploring Thrax phage interaction with host receptors

**Fig. 8:**
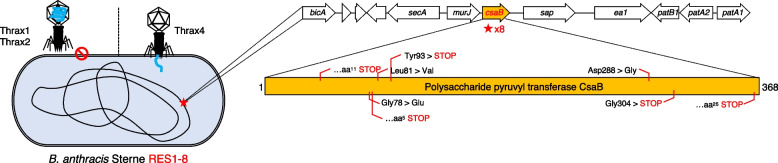
Some Tyroviruses require an intact S-layer for infection. Screening for *B. anthracis* hosts resistant to Thrax1 infection revealed that mutations in the gene encoding polysaccharide pyruvyl transferase CsaB (*csaB*) confers host resistance to Thrax1-2, but not Thrax4. Eight identified mutations in *csaB* ranged from amino acid substitutions (missense) mutations to premature stop via frameshift and nonsense mutation resulting in reduction in CsaB protein length. Raw mutation data are shown in Supplementary Table 11

The variance observed in the tail proteins of Thrax (and Tyrovirus) phages (Fig. 6, Supplementary Fig. 3) suggested these phages may recognise different host components and/or utilise different modes of infection. To this end, we screened for *B. anthracis* hosts resistant to Thrax1 and Thrax4 lysis to determine host factors critical for Tyrovirus infection. While Thrax1-resistant *B. anthracis* Sterne hosts were readily isolated, we could not isolate hosts stably resistant to infection by Thrax4. Thrax1-resistant hosts were found to be cross-resistant to infection by Thrax2 but remained sensitive to Thrax4 (Fig. 8), which was consistent with tail protein similarity patterns (Fig. 6, Supplementary Fig. 3). Eight Thrax1-resistant *B. anthracis* Sterne isolates (RES1-8) were analysed via Illumina sequencing to determine the resistance genotype and all isolates were found to contain unique SNPs or small deletions appearing within a single open reading frame, the *csaB* gene (Fig. 8; Supplementary Table 11). Three of the eight isolates (RES1, RES2, RES7) contained a SNP causing single amino acid substitutions (missense mutations) such as RES1 causing a G78E substitution (glycine [Gly] to glutamate [Glu]; Fig. 8). It is possible these minor substitutions may have disrupted protein structure, stability or occurred within active sites. RES3 and RES5 isolates contained SNPs leading to nonsense mutations causing truncations at position 93 and 304 of CsaB, respectively (Fig. 8). Finally, isolates RES4, RES6 and RES8 each contained a single nucleotide insertion/deletion leading to frameshift and introduction of stop codons (between 5–44 amino acids downstream of the mutation site) throughout *csaB* (Fig. 8).

Polysaccharide pyruvyl transferase CsaB is essential for correct assembly of the S-layer in gram-positive species, such as *B. anthracis*, [54] where it functions in the attachment of proteins containing S-layer homology (SLH) domains to peptidoglycan-associated polysaccharides [55]. *B. anthracis* strains with Δ*csaB* or *csaB* mutants are unable to retain S-layer proteins, such as Sap and EA1, in the cell wall [54, 55]. Mutations in *csaB* have also been shown to abolish the sensitivity of *Bacillus* phage AP50 to infection in *B. anthracis* [56]. In the case of AP50 phage, CsaB was not suggested to be the receptor, but instead proteins assembled onto the S-layer such as Sap and EA1 [56]. Conversely, *Bacillus* phage γ is insensitive to alterations to the S-layer and instead requires the cell surface protein GamR for infection [57]. Interestingly in both the AP50 phage and our work, all mutations mapped to the *csaB* gene suggesting hypermutability of this gene. Overall, resistance of these strains to Thrax1 and Thrax2 infection indicate these phages require an intact S-layer of the cell wall for attachment and infection. Conversely, Thrax4 unaffected by *csaB* mutations indicates it utilises different receptor and/or attachment mechanism. We believe this divergence in *csaB*-sensitivity may extend to other Tyroviruses, with phage tail fiber sequence identity a possible indicator of the mechanism of host-phage interaction (Supplementary Fig. 3).

## Conclusions

This study describes the delineation of a new group of *Bacillus* phages known as the Tyroviruses, named so due to the conservation of three tyrosine recombinases present in the genomes of each member phage. Tyroviruses have been identified around the world, mostly in soil environments, and infect multiple *Bacillus* species including known human pathogens *B. anthracis* and *B. cereus*. Comprehensive genomic analysis describes the organisation of their large genomes (~ 160–170 kb) and details the function of proteins involved in phage DNA replication, repair, virion morphogenesis and host cell lysis. From the perspective of the *B. anthracis* phages isolated here, Thrax1-5, we show that Tyroviruses appear to rely on divergent host interaction and infection pathways, with a subset of the group requiring the host S-layer for successful infection. Ultimately, the expansion of this once-singleton phage group to the current 26 Tyrovirus members highlights the rapidly evolving landscape of phage genomics and sets a platform for the deposition of future Tyroviruses to further unravel the global spread and evolutionary history of these *Bacillus* phages.

## Materials and methods

### Bacterial strains and media

Strains were grown in LB broth (10 g l^−1^ tryptone, 5 g l^−1^ yeast extract, 10 g l^−1^ sodium chloride) and LB agar (with added 12 g l^−1^ agar) at 30 °C. Media components were sourced from ThermoFisher Scientific, Australia.

### Enrichment and isolation of Thrax phages

Soil samples were collected from multiple sites (-12.434, 130.909; -12.362, 130.868; -12.445, 130.932) in the Northern Territory, Australia in April, 2016. Pooled samples [3] from each site were transferred to a 50 ml conical tube, to which 20 ml of nutrient broth was added. Tubes were incubated at 37 °C with shaking at 180 rpm for 2 h. Each tube was centrifuged at 4,600 × g for 20 min. Supernatants were filtered through a 0.22 μm membrane filer (Millipore, Australia). Approximately 20 ml filtrate was concentrated using a 10 kDa molecular weight cut-off filter (Sigma, Australia) down to 1–2 ml. An equal volume of sterile glycerol was then added and tubes stored at -80 °C until required.

The frozen filtrates were thawed and 100 µl of each filtrate was aliquoted on *B. anthracis* Stern lawn plates and allowed to dry. The plates were incubated overnight at 30 °C and inspected for the presence of plaques. Plaques were isolated and resuspended in 500 µl of LB broth and serially diluted and plated on lawn plates. Single plaque purification was performed through five rounds of purification to ensure that each plaque resulted from a single virion.

### Transmission electron microscopy

Carbon coated grids (ProSciTech) were subject to a glow discharge treatment for 60 s and loaded with 5 μl of purified phage filtrate. After 30 s adsorption, the grid was rinsed twice with 5 μl MilliQ H2O and then negatively stained with 3 μl of 2% (w/v) uranyl acetate and left to dry for 30 min. Prepared samples were imaged using a JEM-2100 electron microscope (JEOL).

### Genome sequencing and assembly

Genomic DNA from 1 ml phage filtrate (> 1 × 10^10^ PFU ml^−1^) were extracted using a phenol:chloroform based extraction method. First, filtrates were treated with 10 µg ml^−1^ DNase I, 10 µg ml^−1^ RNase A and 5 mM MgCl_2_ for 30 min at room temperature to remove host DNA and RNA contaminants. Phage virions were precipitated by the addition of 40 mM ZnCl_2_ and incubated for 5 min at 37 °C. Virions were pelleted by centrifugation at 10,000 × g for 5 min, and the supernatant discarded. Precipitated virions were resuspended in 500 µl phage extraction buffer (400 mM NaCl, 20 mM EDTA, 0.5% (w/v) SDS and 50 µg ml^−1^ proteinase K) and incubated for 1 h at 55 °C. An equal volume of phenol:chloroform:isoamyl alcohol (25:24:1) was added to the cooled solution, briefly vortexed, and centrifuged at 16,000 × g for 3 min. The top aqueous layer was transferred into a separate tube to which an equal volume of isopropanol was added. Following incubation on ice for 30–60 min, the mixture was centrifuged at 16,000 × g for 10 min at 4 °C. The supernatant was discarded, and the pellet was subsequently washed in 70% ethanol. After air-drying, the DNA pellet were resuspended in 10 mM Tris–HCl (pH 8.5). Isolated phage DNA (100 ng) were prepared using the NEBNext® Ultra™ II DNA Library Prep Kit (NEB) followed by whole genome sequencing on an Illumina MiSeq v3 600-cycle kit with 300 bp paired-end reads. Raw data were filtered using Trim Galore v0.6.4 with the default settings (Q scores of ≥ 20, with automatic adapter detection) [58]. Phage genomes were assembled with SPAdes v3.9.0 with default settings [59].

### Retrieval of phage genomes similar to Thrax1-5 and Tsamsa phages

Whole genome MEGABLAST against the GenBank nucleotide collection and the Bacillus Phage Database (http://bacillus.phagesdb.org) using Thrax1-5 and Tsamsa phage, in combination with analysis of published literature, was used to identify laboratory isolated Tyrovirus genome sequences. Accession numbers are shown in Table 1. For Tyroviruses obtained from metagenome datasets, the IMG-VR v3 [22] (https://img.jgi.doe.gov/vr/) viral sequence database was queried by BLASTn (e-value 1e^−5^) using the Thrax1-5 and Tsamsa genomes [60]. Matching genome sequences within ~ 15% of the average (~ 165 kb) Tyrovirus genome size (approximately ~ 140–190 kb) were imported into Geneious Prime v2022.2.1 (Biomatters). Genes were first predicted with Glimmer3 [61] and then annotated using the ‘Annotate from…’ function with Thrax1-5 and Tsamsa as references using a 25% similarity threshold. Genomes that were highly annotated (indicating similarity to Thrax1-5 and Tsamsa phages) were retrieved (9 genomes) of which two were removed due to high identity to each other (> 99.5%) resulting in the identification of 7 metagenomic Tyroviruses (Scaffold ID and IMG Genome ID are shown in Table 1). The genomes were annotated with Prokka v1.14.6 using the PHROG database HMM (http://millardlab.org/2021/11/21/phage-annotation-with-phrogs/) [62]. Tyrovirus-like prophages were identified by MEGABLAST using Thrax1 against the GenBank nucleotide collection and whole-genome shotgun contigs collection. The coordinates of the Tyrovirus-like prophage region are approximately 888,188–1,046,443 in *Bacillus velenzensis* strain FIO1408 (CP061938), 364,192–508,829 in *Bacillus licheniformis* YNP3-TSU (MEDC01000003) and 633,599–711,080 in *Bacillus sonorensis* J41TS (BORD01000002).

### Phylogenetic analysis

For VIRIDIC phage similarity analysis, phage genome nucleotide sequences were analysed using VIRIDIC v1.0 (http://rhea.icbm.uni-oldenburg.de/VIRIDIC/) [23].

For vConTACT2 reticulate network analysis, Tyrovirus phage genomes were annotated with Prokka v1.14.6, and the phage protein sequences combined with phage protein sequences from all complete phage genomes present on Genbank in April 2022 (17,001 genomes) as generated by inphared [63]. Gene2Genome was used to assign and map protein coding sequences prior to the use of vConTACT2 v0.9.19 using default settings. The network was visualised with Cytoscape v3.9.1 [64] using the default layout method for the entire network and then using an edge-weighted spring-embedded model in the zoom view to better understand grouping characteristics.

For VICTOR analysis, Tyrovirus phage protein sequences were obtained from Prokka as above and analysed using the VICTOR3 pipeline (https://ggdc.dsmz.de/victor.php) [25]. Briefly, pairwise comparisons of the amino acid sequences were conducted using the Genome-BLAST Distance Phylogeny (GBDP) method [65] under settings recommended for prokaryotic viruses [25]. The resulting intergenomic distances were used to infer a balanced minimum evolution tree with branch support via FASTME including SPR postprocessing [66]. Branch support was inferred from 100 pseudo-bootstrap replicates each. Taxon boundaries at the species, genus and family level were estimated with the OPTSIL program [67], the recommended clustering thresholds [25] and an F value (fraction of links required for cluster fusion) of 0.5 [68]. Phylogenetic trees inferred using the D6 formula (optimised for amino acids) were displayed using iTOL v5 (https://itol.embl.de) [69].

Phylogenetic reconstruction of Tyroviral tail fibers was performed with Phylogeny.fr (phylogeny.fr) [70] using MUSCLE v3.8.31 alignment [71] and PhyML v3.1 using the maximum likelihood method for phylogenetic reconstruction [72].

### Core genome analysis

Core protein analysis was performed using the Roary pipeline. Briefly, Tyrovirus phage genomes were annotated with Prokka as above with the GFF3 output format used downstream. Roary v3.13.0 core gene alignment [73] was employed with a reduced BLASTp identity threshold of 30%, as suggested previously [26]. The representative core proteome (Thrax1 phage) was subject bioinformatic analysis (as described in section below) to determine protein function. Charts were generated with Prism v9.0 (Graphpad).

### Genome annotation

For the genome termini analysis, the Thrax1-5 assembled genomes, and raw sequence data, were subject to PhageTerm v1.0.12 analysis on Galaxy Pasteur (https://galaxy.pasteur.fr). Raw sequence reads were then manually inspected in CLC Genomics WorkBench v9.5.5 (Qiagen) using the duplicated sequence’s function to detect regions of high starting position coverage to confirm the PhageTerm results. Short direct terminal repeat (DTR) sequences in other Tyroviruses were identified using the ‘Annotate from…’ function with DTRs identified in Thrax1-5 and Tsamsa as references using a 50% similarity threshold using Geneious Prime v2022.2.1 (Biomatters). Alignments between DTRs were performed using the Geneious alignment tool with default settings. For genome annotation, assembled Thrax1-5 genomes were imported into Geneious Prime v2022.2.1 (Biomatters). Genes were predicted with Glimmer3 [61] and then manually inspected for the presence of ribosome binding sites (RBS). ORFs were annotated using a combination of the NCBI Conserved Domain Database (CDD) [31], profile Hidden Markov model (HMM) similarity using HHpred [32] and the Virfam webserver [35]. tRNAs were identified using tRNAscan-SE v2.0 [74] and Aragorn v1.2.41 [75]. Figures were generated using CLC Genomics WorkBench v9.5.5 (Qiagen), shinyCircos v1.6.0 [76] and Clinker v0.0.12 [77]. Amino acid similarity calculations were performed using Clustal Omega v1.2.3 [78] in Geneious Prime v2022.2.1 (Biomatters) with the Blosum 62 similarity matrix. Average amino acid similarity values presented in the text refer to the average value of all similarity values in the matrix. Raw data and calculations are available in Supplementary Table 12.

### Generation and analysis of Thrax mutants

Lawn plates of *B. anthracis* Sterne were prepared and Thrax1 and Thrax4 were flooded in high titre and grown at 30 °C overnight. Colonies that emerged in the clearing were picked and streaked out for a total of three times to ensure a pure isolate. Thrax1, Thrax2 and Thrax4 phages were then spotted on lawn plates of these isolates to test the ability for phage infection. DNA was extracted using the Wizard Genomic DNA Purification Kit (Promega) and DNA were prepared for Illumina sequencing as above. The wildtype *B.* anthracis Sterne was also prepared, sequenced and assembled with Unicycler v.0.4.8 using the default settings [79]. Raw data were filtered using Trim Galore as above and SNP analysis was performed with Snippy v4.6.0 using default settings (https://github.com/tseemann/snippy). Background mutations identified in the mutant isolates, due to genetic drift or minor error in the reference assembly, were screened out through SNP comparison between all mutant isolates (i.e. all mutant isolates equally contained X mutation in Y position), with remaining unique mutations deemed biologically relevant. All identified mutations (background and unique) are listed in Supplementary Table 11.

## Supplementary Information

**Additional file 1: Supplementary Table 1.** Thrax phage host range on virulent (capsulated) *B. anthracis *strains. **Supplementary Table 2.** VIRIDIC intergenomic similarity raw data. **Supplementary Table 3.** vConTACT2 viral cluster raw data. **Supplementary Table 4.** Tyrovirus core proteome list. **Supplementary Table 5.** *Bacillus* phage Thrax1 genome annotations. **Supplementary Table 6.*** Bacillus* phage Thrax2 genome annotations. **Supplementary Table 7.*** Bacillus* phage Thrax3 genome annotations. **Supplementary Table 8.*** Bacillus* phage Thrax4 genome annotations. **Supplementary Table 9.*** Bacillus* phage Thrax5 genome annotations. **Supplementary Table 10.** *attP *site sequence conservation across Tyroviruses. **Supplementary Table 11.** Thrax1-resistant *B. anthracis* Sterne strain raw mutation data. **Supplementary Table 12.** ClustalW protein sequence similarity matrices and average calculations.

**Additional file 2:** **Supplementary Figure 1.** The Tyroviral core proteome. **Supplementary Figure 2.** The short direct terminal repeat (DTR) sequence in Tyroviruses. **Supplementary Figure 3.** Tyroviral tail fibers phylogenetically separate based on host species. **Supplementary Figure 4.** Tyro6-7 metagenomic phages and Tyrovirus-like prophages in Bacillus species lack the plasmid maintenance region.

## Data Availability

The phage genomes sequenced and assembled in this study are available on NCBI GenBank under the following accession numbers: *Bacillus* phage vB_BanS_Thrax1, ON548417; *Bacillus* phage vB_BanS_Thrax2 ON548418; *Bacillus* phage vB_BanS_Thrax3 ON548419; *Bacillus* phage vB_BanS_Thrax4 ON548420; *Bacillus* phage vB_BanS_Thrax5 ON548421. All data generated or analysed during this study are included in this published article [and its supplementary information files].
