# Supplementary material for: Tyroviruses are a new group of temperate phages that infect Bacillus species in soil environments worldwide
Source: BMC Genomics. 2022 Nov 28;23:777. doi: 10.1186/s12864-022-09023-4 (PMC9703825; doi:10.1186/s12864-022-09023-4)
Supplement: Supplementary file 2 — Additional file 2: Supplementary Figure 1. The Tyroviral core proteome. Supplementary Figure 2. The short direct terminal repeat (DTR) sequence in Tyroviruses. Supplementary Figure 3. Tyroviral tail fibers phylogenetically separate based on host species. Supplementary Figure 4. Tyro6-7 metagenomic phages and Tyrovirus-like prophages in Bacillus species lack the plasmid maintenance region. [file 12864_2022_9023_MOESM2_ESM.pdf]

Supplementary Figure 1

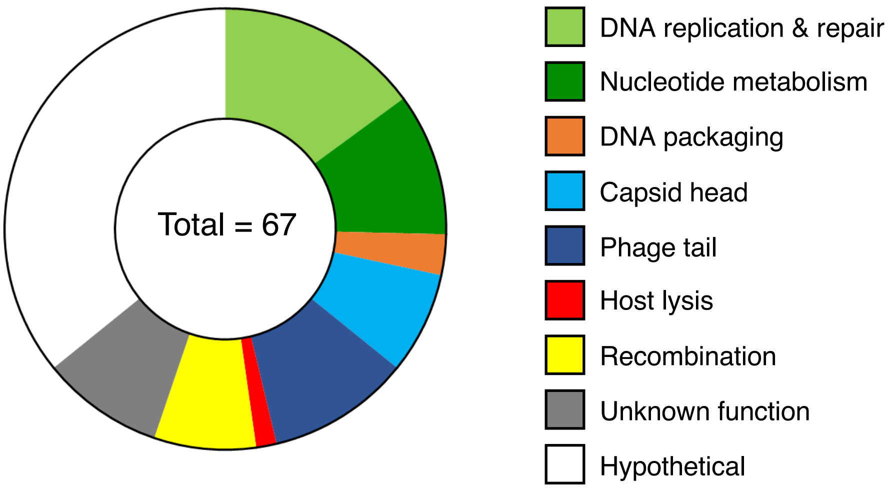

Supplementary Figure 1 I The Tyroviral core proteome. Core protein analysis of 26 Tyrovirus genomes including Thrax1-5. The classification of 67 proteins that were identified in common in all Tyroviral genomes is shown. The list of 67 proteins is shown in Supplementary Table 4.

Supplementary Figure 2

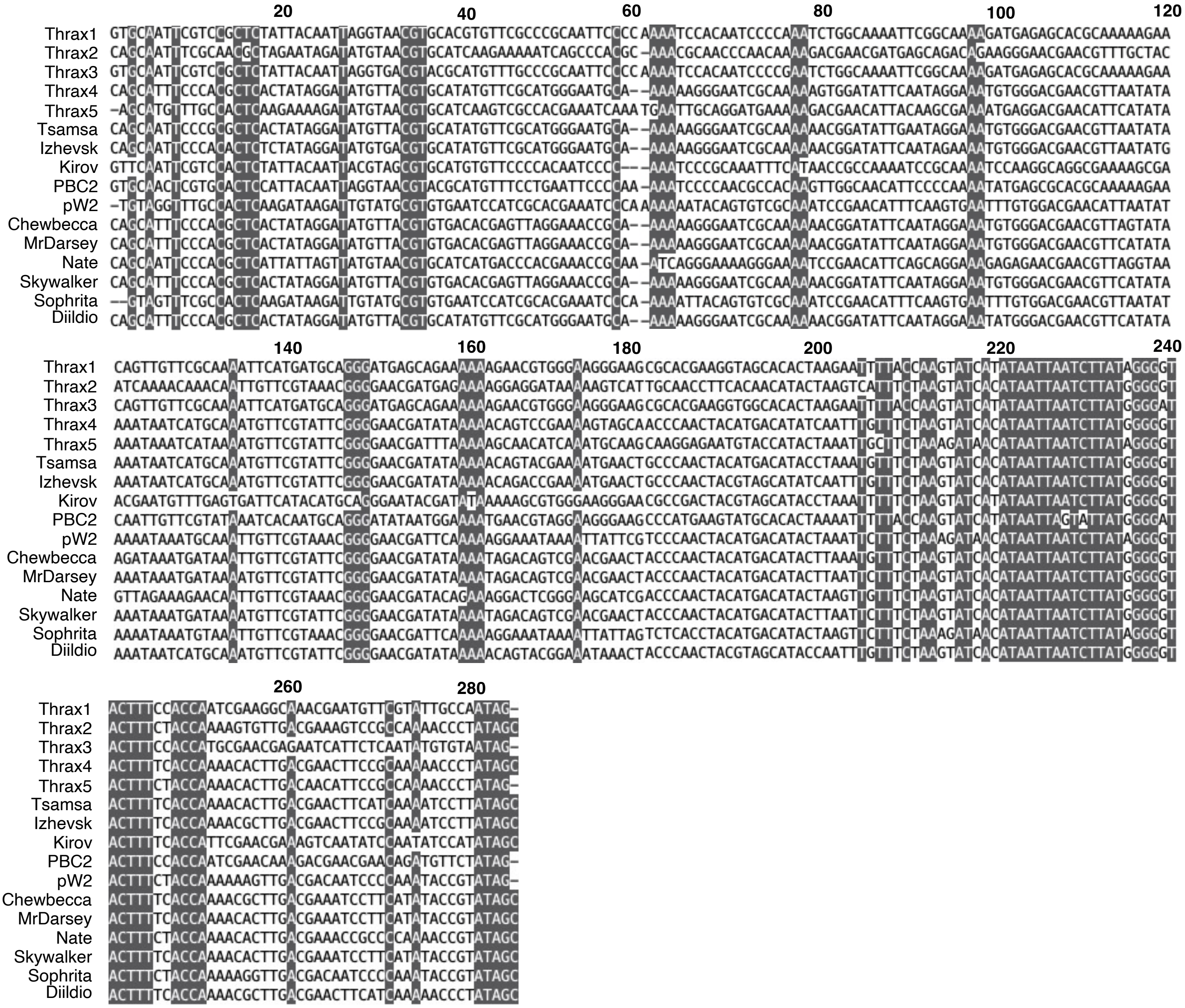

Supplementary Figure 2 | The short direct terminal repeat (DTR) sequence in Tyroviruses. DTR sequences were identified either via empirical means (i.e. Sanger sequencing in Tsamsa or analysis of raw sequencing data in Thrax1-5) and used to identity DTRs in other Tyroviruses by sequence comparison. Alignment of DTRs shows some regions of high sequence identity. Size markers represent nucleotides.

Supplementary Figure 3

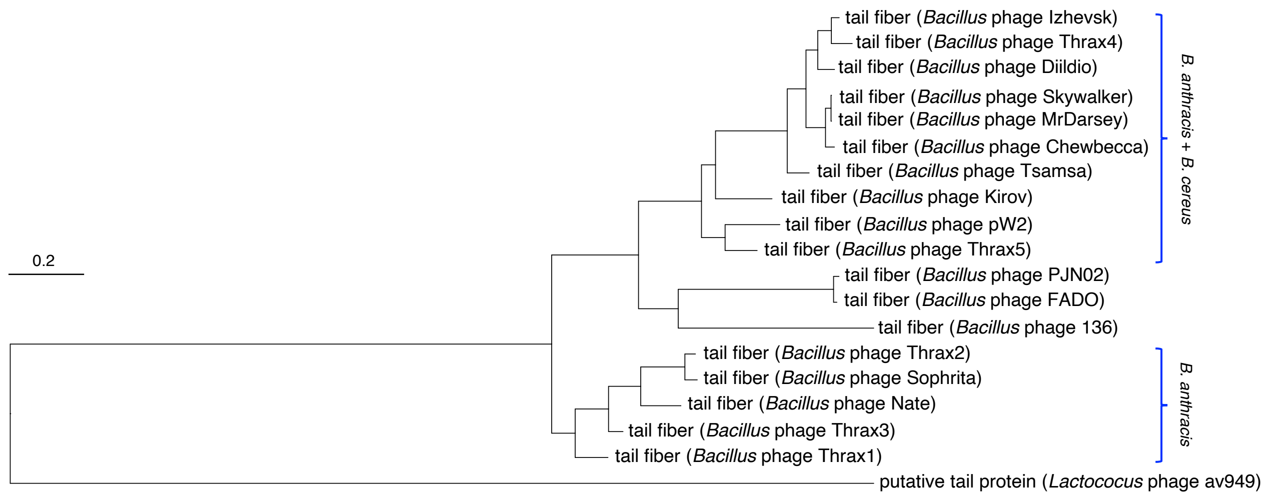

Supplementary Figure 3 I Tyroviral tail fibers phylogenetically separate based on host species. Phylogenetic reconstruction based on tail fiber protein sequence from 19 Tyroviral phages demonstrating clustering using the maximum likelihood method. *Lactococcus* phage av949 as used as an out-group. Scale bar represents the number of amino acid substitutions per site.

Supplementary Figure 4

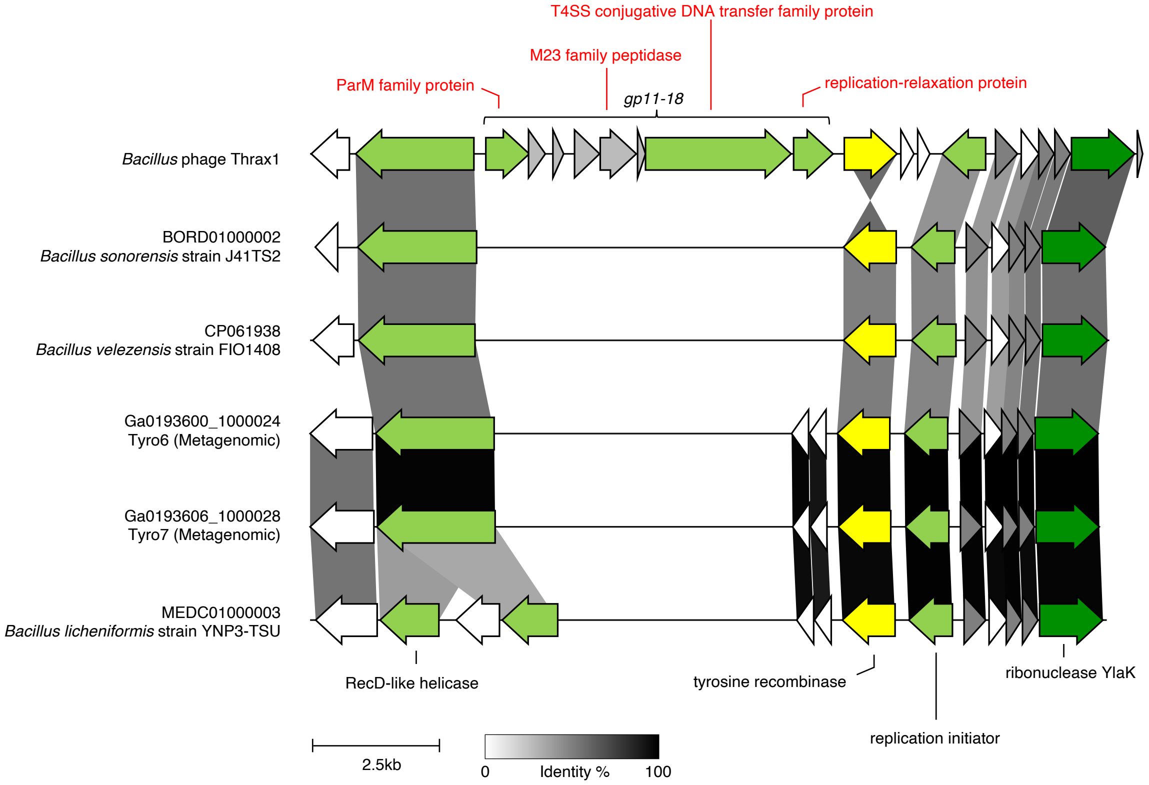

Supplementary Figure 4 I Tyro6-7 metagenomic phages and Tyrovirus-like prophages in *Bacillus* species lack the plasmid maintenance region. Alignment between regions in proximity to *gp11-18* of *Bacillus* phage Thrax1 (coordinates 3,160-19,208), *Bacillus sonorensis* strain J41TS2 (BORD01000002, coordinates 687,704-697,675) *Bacillus velezensis* strain FIO1408 (CP061938, coordinates 947,668-957,592), Tyro6 (Metagenomic, Ga0193600_1000024, coordinates 108,311-117954), Tyro7 (Metagenomic, Ga0193606_1000028, coordinates 22,063-31,725) and *Bacillus licheniformis* strain YNP3-TSU (MEDC01000003, coordinates 417,401-428,333) and illustrates the addition of *gp11-gp18* plasmid maintenance genes in Thrax1 in between the RecD-like helicase and tyrosine recombinase genes. The tyrosine recombinase occurs in the opposite direction in Tyro6-7 and prophage sequences. A gene insertion in the RecD-like helicase gene in *B. licheniformis* strain YNP3-TSU is also noted. Gene color scheme is consistent with that described in Figure 5. Text in red indicates differences between aligned sequences. Gene identity is shown by greyscale shading. Scale bar represents 2.5 kb.
